# Nanomaterial cytotoxicity is composition, size, and cell type dependent

**DOI:** 10.1186/1743-8977-7-22

**Published:** 2010-08-21

**Authors:** Syed K Sohaebuddin, Paul T Thevenot, David Baker, John W Eaton, Liping Tang

**Affiliations:** 1 Department of Bioengineering, University of Texas at Arlington, Arlington, TX, USA; 2 Molecular Targets Program, James Graham Brown Cancer Center, University of Louisville, Louisville, KY, USA

## Abstract

**Background:**

Despite intensive research efforts, reports of cellular responses to nanomaterials are often inconsistent and even contradictory. Additionally, relationships between the responding cell type and nanomaterial properties are not well understood. Using three model cell lines representing different physiological compartments and nanomaterials of different compositions and sizes, we have systematically investigated the influence of nanomaterial properties on the degrees and pathways of cytotoxicity. In this study, we selected nanomaterials of different compositions (TiO_2 _and SiO_2 _nanoparticles, and multi-wall carbon nanotubes [MWCNTs]) with differing size (MWCNTs of different diameters < 8 nm, 20-30 nm, > 50 nm; but same length 0.5-2 μm) to analyze the effects of composition and size on toxicity to 3T3 fibroblasts, RAW 264.7 macrophages, and telomerase-immortalized (hT) bronchiolar epithelial cells.

**Results:**

Following characterization of nanomaterial properties in PBS and serum containing solutions, cells were exposed to nanomaterials of differing compositions and sizes, with cytotoxicity monitored through reduction in mitochondrial activity. In addition to cytotoxicity, the cellular response to nanomaterials was characterized by quantifying generation of reactive oxygen species, lysosomal membrane destabilization and mitochondrial permeability. The effect of these responses on cellular fate - apoptosis or necrosis - was then analyzed. Nanomaterial toxicity was variable based on exposed cell type and dependent on nanomaterial composition and size. In addition, nanomaterial exposure led to cell type dependent intracellular responses resulting in unique breakdown of cellular functions for each nanomaterial: cell combination.

**Conclusions:**

Nanomaterials induce cell specific responses resulting in variable toxicity and subsequent cell fate based on the type of exposed cell. Our results indicate that the composition and size of nanomaterials as well as the target cell type are critical determinants of intracellular responses, degree of cytotoxicity and potential mechanisms of toxicity.

## Background

In recent years, a wide range of nanomaterials have been developed for various applications in industrial, electrical, agricultural, pharmaceutical and medical fields due to their unique properties [1]. Increasing evidence suggests that the special physicochemical properties of these nanomaterials pose potential risks to human health [2]. Therefore, considerable effort has been placed on identifying the potential toxicity of nanoparticles to cells and organisms. Many groups have reported that nanoparticles of different compositions do indeed present toxicological concerns. For example, exposure of cells and animals to quartz and mineral dust particles (e.g., coal and silicates), asbestos fibers, and experimental instillation of TiO_2 _and carbon black nanoparticles in animal lungs induce oxidative injury, inflammation, fibrosis, cytotoxicity, and release of pro-inflammatory mediators [1,3-5]. In addition, animal studies have confirmed an increase in pulmonary inflammation, oxidative stress and distal organ involvement upon respiratory exposure to nanoparticles [6-8].

*In vitro *studies have generally supported the pathophysiological responses found in animal models, including increased generation of ROS in cells exposed to various nanomaterials [9-12]. Many *in vitro *studies have identified increased ROS generation as an initiating factor of toxicity in nanoparticle exposed cells [13,14]. However, it has been difficult to establish a comprehensive mechanism of nanoparticle cytotoxicity based on previous, and rather inconsistent, observations. For instance, some reports indicated that exposure of cells to TiO_2 _leads to lipid peroxidation, DNA damage, caspase activation followed by micronuclei formation, chromatin condensation and eventual cell death via apoptosis [14-17]. However, other investigators have reported that TiO_2 _nanoparticle exposure instead causes plasma membrane damage and decrements in mitochondrial function [18-20]. There are even reports that TiO_2 _exposure does not lead to membrane damage, caspase activation or cell death [21,22]. In addition, cells exposed to silicon oxide (SiO_2_) nanoparticles show caspase activation and cell death via apoptosis but the suggested pathways leading to apoptosis differed [23-25]. One study concluded that lysosomal destabilization was the initiating factor [24] whereas two others concluded that loss of mitochondrial membrane integrity was the predominant cause of cell death [23,25]. In fact, it is possible that lysosomal destabilization and loss of mitochondrial membrane integrity may be related events [26]. In contrast to these findings, other investigators reported that SiO_2 _exposure leads to plasma membrane damage [9,27] similar to observations with MWCNTs [28]. However, other studies indicated that MWCNT induced cell cycle arrest and caused cell death via apoptosis [29,30]. MWCNT were also reported to cause a decrease in mitochondrial membrane potential [31,32] whereas another study reported that these nanotubes were non-toxic [33].

These conflicting results are likely caused by variations in experimental procedures, composition and size of nanoparticles employed and the use of varying cell types. Further differences such as protein adsorption prior to cell exposure and particle dispersion/agglomeration have also been recently shown to play important roles. These input variables are likely related to varied toxicological outputs. Although it seems logical that different cell types will respond differently to nanomaterials, this subject has only recently been explored [29,34-36]. It is of paramount importance to identify the mechanistic response of exposure-prone cells to nanomaterials as they are not only potential environmental exposure hazards [37,38], but are continuously employed in biomedical applications in many different tissues and compartments inside the body [39,40]. Therefore it is necessary that we both understand how different cells respond to nanomaterials, and through what mechanisms. This information would help in the development of nanoparticles with improved safety and efficacy.

We have therefore carried out a systematic and comparative study on the cytotoxic effects of common, widely used nanomaterials of varying composition (TiO_2_, SiO_2_, MWCNT) and size (MWCNT: < 8 nm, 20-30 nm, > 50 nm). Three cell types were chosen for this investigation based on their physiological function and location. 3T3 fibroblasts were chosen as a model for stromal cells, which can be found in matrix and connective tissue throughout the body. For the second cell type, we chose telomerase immortalized human bronchiolar epithelial cells (hT) as a model cell type for inhalation exposure to nanomaterials. The third cell type was RAW 264.7 macrophages, which are commonly included in nanomaterial toxicity investigations as an inflammatory cell type. The effects of these nanomaterials on cytotoxicity, protein adsorption, cellular uptake, ROS generation, lysosomal stability, mitochondrial activity, activation of caspase 3 and 7 and mode of cell death (apoptosis vs. necrosis) were investigated. The role of material composition was investigated using nanomaterials of similar diameters but different compositions. The effects of MWCNT of varying diameter but similar length on cell toxicity were also evaluated.

## Methods

### Reagents

Dulbecco's Modified Essential Media (DMEM) was purchased from Sigma-Aldrich (St. Louis, MO). Fetal Calf Serum (FCS) and antibiotics were obtained from Atlanta Biologicals (Lawrenceville, GA). Bronchial epithelial cell growth medium (BEGM) was purchased from Lonza (Basel, Switzerland). 3-(4,5-dimethylthiazol-2-yl)-5-(3-carboxymethoxyphenyl)-2-(4-sulfophenyl)-2H-tetrazolium, inner salt (MTS) was procured from Promega Corporation (Madison, WI). 2',7'-dichlorodihydrofluorescein diacetate (H_2_DCFDA) was purchased from Invitrogen (Carlsbad, CA). Acridine Orange (AO) and Sensolyte Homogeneous AMC Caspase-3/7 Assay Kit were acquired from Merck (Darmstadt, Germany) and Anaspec (San Jose, CA), respectively. MitoProbe DilC_1_(5) Assay Kit and propidium iodide were purchased from Invitrogen (Carlsbad, CA). BCA protein assay kit was from Pierce (Rockford, IL). Annexin V-FITC Apoptosis Kit was purchased from Biovision, Inc. (Mountain View, CA).

### Test materials

To assess the influence of material chemical composition on nanoparticle-mediated cell toxicity, we selected TiO_2 _(anatase; 5-10 nm in diameter) and SiO_2 _(30 nm in diameter). We also employed MWCNTs (0.5-2 μm length) with three different ranges of diameters, < 8 nm, 20-30 nm, and > 50 nm to examine the influence of material size on nanoparticle-induced cell toxicity. All nanomaterials were acquired from Sun Innovations, Inc. (Fremont, CA). The physical properties of the nanomaterial test groups have been summarized as provided by the manufacturer (Table 1).

**Table 1:** Nanomaterial Physical Characteristics.

Description	Purity	Diameter	Length	Special Surface Area	Particle Form	True Density
Titanium Oxide	99%	5-10 nm	n/a	215 m^2^/g	Crystal Phase: Anatase	3.9 g/cm^3^

Silicon Oxide	99.6%	30 nm	n/a	165 m^2^/g	UV Diffraction: > 75%	> 0.11 g/cm^3^

MWCNT < 8 nm	> 95%	ID*-2-5 nm OD*- < 8 nm	.5-2 μm	40-300 m^2^/g	Amorphous Carbon	2.1 g/cm^3^

MWCNT 20-30 nm	> 95%	ID*-5-10 nm OD*-20-30 nm	.5-2 μm	> 110 m^2^/g	Amorphous Carbon	2.1 g/cm^3^

MWCNT > 50 nm	> 95%	ID*-5-15 nm OD*- > 50 nm	.5-2 μm	> 40 m^2^/g	Amorphous Carbon	2.1 g/cm^3^

The dimensions and physical properties were characterized by TEM, for TiO_2 _and SiO_2_, and by TEM, X-Ray Diffraction and Raman Spectroscopy for MWCNTs. MWCNTs of different dimensions were manufactured via Carbon Vapor Deposition (CVD). This data was provided by the nanomaterial manufacturer. ID - Inner Diameter; OD - Outer Diameter.

### Characterization of nanoparticles in physiological solution and cell culture media

The behaviour of nanoparticles in suspension was analyzed using dynamic light scattering (DLS). The measurements were made using Zetapals (Brookhaven Instruments Corp.). All nanomaterials used in this investigation were suspended in PBS and in culture media containing serum at 10, 100 and 1000 μg/ml and sonicated for 60 sec at 40W in an ice bath to disperse the nanoparticles and stored overnight. Nanoparticles were diluted to 1 μg/ml into distilled water, sized and tabulated as the average nanoparticle size. Prior to sizing, the nanoparticles were put through another sonication cycle for dispersion and diluted to 1 μg/ml in deionized water for testing.

BCA assays were performed to analyze the adsorption of protein from cell culture media in the absence of cells as previously described [41]. Culture media containing 10% FCS was prepared with 100 μg/ml nanoparticle suspensions, sonicated as mentioned above and incubated for periods of 30 min (maximum protein adsorption) [41,42] and 2 hours (reversible protein exchange with culture media) [43]. After incubation, nanoparticle suspensions were centrifuged and the pellets assayed to determine protein depletion from the culture media with nanoparticle free culture media as control. Optical density was measured using a SpectraMAX 340 (Molecular Devices, Sunnyvale, CA).

### Cell culture

3T3 fibroblasts and RAW 264.7 macrophages were obtained from ATCC (Manassas, VA). 3T3 cells were cultured in DMEM supplemented with 10% FCS and 1% antibiotics (10,000 units/ml penicillin and 10,000 μg/ml streptomycin in 0.85% saline). As a model of a respiratory cell type, we used telomerase-immortalized human bronchiolar epithelial cells (hT), a gift from Dr. B. J. Rollins (Harvard Medical School, Boston, MA) and were cultured in bronchial epithelial cell complete growth medium (BEGM) as previously described [44]. Unless stated otherwise, for all experiments, cells were plated in 96-well plates at a density of 5000 cells per well in 100 μl of culture medium. Cells were allowed to attach and achieve approximately 80% confluence prior to starting the experiments. The particle suspensions were prepared in complete culture media consisting of DMEM supplemented with 10% FCS and 1% antibiotic solution. The particle suspensions were sonicated and briefly vortexed to resuspend the particles prior to cell culture study. The particle suspension was sonicated using an ultrasonic tip at 30% amplitude for 3-5 seconds.

### Cellular uptake or interaction with nanoparticles

The degree of nanoparticle uptake or adsorption on cellular membranes was examined by analyzing forward scatter (FSC) verses side scatter (SSC) using flow cytometry (Becton Dickinson, FACSArray Bioanalyzer System, San Jose, CA) as described previously [45]. Briefly, cells were incubated with 100 μg/ml of TiO_2_, SiO_2_, MWCNT < 8 nm, MWCNT 20-30 nm and MWCNT > 50 nm for 3 hours in the appropriate culture media to allow sufficient time for cellular uptake of nanomaterials based on our recent and other publications [19,45]. Following this, the cells were detached by cell scraping and suspended in PBS for flow cytometry. Following gating, control and nanoparticle-exposed cells were run and plotted to examine increase in side scatter (SSC) due to endocytotic or adsorptive nanoparticle interaction.

### Cytotoxicity

The effects of nanomaterials on the viability of 3T3, hT, and RAW cells were evaluated using MTS [3-(4,5-dimethylthiazol-2-yl)-5-(3-carboxymethoxyphenyl)-2-(4-sulfophenyl)-2H-tetrazolium] reduction [19]. MTS was chosen for this study due to the solubility of the reduced product in culture medium and because MWCNT interfere with the MTT reduction assay [46,47]. Briefly, culture medium from the wells was removed and replaced with 100 μl new culture media containing particles at concentrations of 10, 100, and 1000 μg/ml. Consistent with previous observations [15,17,20], we found 24 hours to be the optimal time for measurements of the effects of nanoparticles on cell survival. After 24 hours of exposure, the culture medium was removed and the cells were rinsed three times in PBS to remove free nanomaterials. After two hours of incubation with MTS solution, the supernatant was transferred to a new 96 well plate to ensure that light transmission was not disturbed by nanomaterials incorporated into cells. Viability of the adherent cells was then determined using MTS reduction. Each treatment was carried out in quadruplicate and cell survival was calculated with respect to untreated controls. Viability of 3T3 fibroblasts was also assessed every 6 hours for 24 hours using MTS reduction.

Prior to assay, MTS solution was incubated with 10 μg/ml or 100 μg/ml of each test group using identical protocols detailed for cell culture. There was very little to no change in absorbance values and these changes were insignificant from MTS-culture media controls. Coupled with PBS washing to remove excess nanoparticle prior to adding the MTS reagent mixture, we find no detectable nanoparticle interference with the MTS assay.

### Intracellular generation of ROS

Cellular generation of ROS was determined using oxidation of 2',7'- dichlorodihydrofluorescein diacetate (H_2_DCFDA) as previously described [31]. Specifically, culture media from the wells was removed and replaced with 100 μl new culture media containing particles at a concentration of 100 μg/ml. After two hours of incubation with nanomaterials, a time sufficient to stimulate intracellular ROS production [31], the cells were rinsed three times with PBS to remove unbound nanomaterials. The cells were then incubated with 100 μl of H_2_DCFDA (5 μM in PBS) at 37°C for 30 min. Following this, the wells were rinsed twice with PBS to remove excess dye and 200 μl of PBS was added to each well. Fluorescence was measured with excitation at 485 nm and emission at 530 nm using a Tecan Infinite M 200 microplate reader (San Jose, CA) with measurements done in quadruplicate. The fluorescence intensities of treated cells were normalized to untreated controls and plotted as Relative Fluorescence Units (RFU).

### Lysosomal membrane integrity

Destabilization of the lysosome has recently been linked to cytotoxicity involving ROS generation and associated disruption of mitochondrial membrane potential [48,49]. Lysosomal membrane stability was assessed using the acridine orange (AO) relocation technique as verified in previous work [50-52]. AO is a metachromatic fluorophore and a lysosomotropic base (pKa = 10.3) which diffuses into cells and accumulates in lysosomes by proton trapping. This accumulation produces a change in the fluorescence emission of the probe (from cytosolic green to red within the lysosomes due to concentration-dependent stacking of the AO). Disruption of the lysosomal membrane can be assessed by measuring the change in intracellular AO fluorescence (i.e., loss of lysosomal red signal and gain of cytoplasmic green) [50-52]. For these tests, cells were grown to sub-confluence on coverslips. The cells were then loaded with AO (5 μg/ml) in culture media for 15 min at 37°C, rinsed with DMEM and incubated with the nanomaterials (final concentration of 100 μg/ml) as reported earlier [23]. After 4 hr incubation, the culture medium containing nanoparticles was removed and the cells were rinsed thrice with PBS. For qualitative studies, the cell images were recorded using a Leica DMLB fluorescence microscope equipped with a Nikon E500 camera (8.4 V, 0.9 A, Nikon Corp., Japan). For quantitative analyses, cells at 80% confluence were incubated with AO as above, rinsed with DMEM and then incubated with 100 μl of nanomaterials (100 μg/ml) for 4 hours at 37°C. Four samples were used in each treatment. After exposure, the cells were rinsed three times with PBS and incubated with 100 μl of PBS prior to fluorescence measurements with excitation at 485 nm emission at 530 nm (green cytoplasmic AO) and 620 nm (red lysosomal AO) using a Tecan microplate reader (Tecan Group Ltd.). The relative lysosomal membrane permeabilization was calculated based on the percent change in the 530 nm/620 nm intensity ratio of treated cells with respect to the controls.

### Mitochondrial membrane potential

Nanoparticle mediated damage to mitochondria was assessed using the MitoProbe DilC_1_(5) (Invitrogen, CA) Assay following manufacturer's instructions. Cells were seeded in 96 well plates as described above and exposed to nanomaterials at a concentration of 100 μg/ml for 6 hrs. After exposure, control and nanomaterial-exposed cells were rinsed twice in PBS and incubated with DilC_1_(5). Cells exposed to carbonyl cyanide 3-chlorophenylhydrazone (CCCP) were used as negative controls (i.e., lacking membrane potential). Cells were then rinsed and suspended in PBS prior to fluorescence measurements with excitation at 638 nm and emission at 658 nm using a Tecan microplate reader. Fluorescence intensity values of treated cells were normalized to untreated controls as plotted as percent change in fluorescence intensity with respect to controls.

### Caspase-3 and Caspase-7 activation

Both caspase-3 and caspase-7 are important in the execution phase of apoptosis [53]. The degree of caspase-3 and caspase-7 activation in cells exposed to nanomaterials was determined using the Sensolyte Homogeneous AMC Caspase-3/7 assay kit (Anaspec, CA) following manufacturer's instructions. Briefly, the assay employs Ac-DEVD-AMC as the fluorogenic indicator of caspase-3/7 activities. The cells were incubated with 100 μl of media containing nanoparticles (100 μg/ml) for 10 hours, a sufficient time for maximum caspase activation as established earlier [13]. Then, 50 μl of the caspase-3/7 substrate was added to each well. Cleavage products were quantified at excitation at 354 nm and emission at 442 nm using a Tecan microplate reader. The fluorescence intensities were normalised with untreated controls and plotted as Relative Fluorescence Units (RFU).

### Annexin V/Propidium iodide assay

To distinguish the mode of nanomaterial induced cell death, annexin V/Propidium iodide assay was performed as previously described [54]. Briefly, 3 × 10^5 ^cells grown on glass cover slips were incubated with media containing nanoparticles (100 μg/ml) for 20 hours. Cell culture media in the absence of nanoparticles was added to control cells. After 20 hours, cells were rinsed twice in PBS, centrifuging the cell suspension before and between washes to prevent loss of any dead or detached cells, and then double stained with Annexin V-FITC and Propidium Iodide according to manufacturer's protocol. The coverslips were inverted on a glass slide and images were analyzed by fluorescence microscopy with Leica DMLB fluorescence microscope equipped with a Nikon E500 camera (Nikon Corp., Japan). Apoptotic and necrotic cell percentages were then calculated for both treated and untreated cells. Cells undergoing apoptosis, both early and late apoptosis, were distinguished from cells undergoing necrosis based on previously published criteria [54,55]. Briefly, cells undergoing apoptosis are Annexin V^+^/PI^-^, cells undergoing late apoptosis are Annexin V^+^/PI^+ ^while cells undergoing necrosis are Annexin V^-^/PI^+^.

### Statistics

The results of cell viability, ROS generation, lysosomal membrane permeability, caspase-3/7 activity, and Annexin V-FITC/PI assay are presented as mean ± standard deviation. Statistical comparisons were conducted using the student's *t*-test and ANOVA in Graph Pad Prism (Graph Pad software, San Diego, CA). Dunnet post tests were used to compare treatment groups to control and Bonferroni tests were used for inter-group comparisons. Differences between different groups were considered statistically significant at p < 0.05.

## Results

### Nanoparticle characterization in solution

Differences in particle dispersion/agglomeration have recently been shown to play important role in nanomaterial toxicity. Therefore we determined each nanomaterial's physical and chemical properties in physiological media including serum containing culture media. Size distribution of nanomaterials was assessed using Dynamic Light Scattering after dispersion in PBS and in culture media with serum (Table 2). Apparent particle size increased in both PBS and culture media. MWCNT > 50 nm at 10 μg/ml quickly dispersed in both, PBS and culture media, and maintained dispersion longer than other nanomaterials tested. However, as the concentration increased from 10 μg/ml to 100 μg/ml this effect was masked. The amount of aggregation of each nanomaterial in PBS when compared to culture media containing serum was identical. Overall, we observed the level of aggregation to be concentration dependent. We also observed a similar size distribution of the nanomaterials (~300-500 nm) at 10 and 100 μg/ml following sonication with the exception to MWCNT > 50 nm which had a size range from 700-800 nm at 100 μg/ml.

**Table 2:** Properties of Nanoparticles in Solution.

Particles	Description	Average Size	Average Size in PBS	Average Size in Culture Media
TiO_2_	Titanium Dioxide (Anantase)	5-10 nm	200-400 nm	350-500 nm
SiO_2_	Silicon Oxide	30 nm	200-400 nm	300-500 nm
MWCNT <8		< 8 nm	180-240 nm	450-550 nm
MWCNT 20-30	MWCNT	20-30 nm	100-350 nm	450-500 nm
MWCNT >50		> 50 nm	70-153 nm*	700-800 nm

Nanoparticles were sized using DLS in PBS and cell culture media with serum. Values were compared with manufacturers' reported particle size. Materials were suspended in solution via sonication and stored overnight before repeat sonication cycles and sizing. Values reported are nanoparticle population range.

* - Less tendency to aggregate in PBS and culture media.

### Nanoparticle interactions with serum proteins

**Figure 1:**
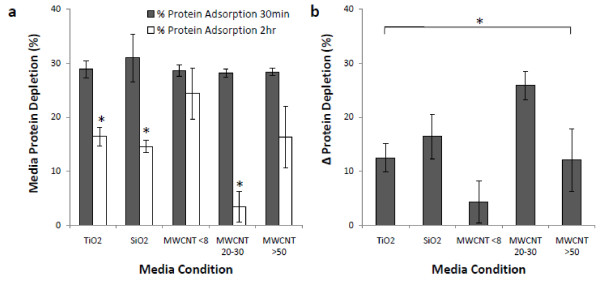
**Nanoparticle mediated protein depletion from cell culture media**. Nanoparticles were incubated in serum containing culture media (10%) for periods of 30 minutes and 2 hours, with the early time point representing time for significant protein interaction and the later time point representing onset of particle uptake. Initially, all nanoparticles adsorb approximately 30% of the serum from the media, followed by a period of particle dependent reversible exchange of protein with culture media (a). Plotting of change in protein depletion from the media reveals significant differences in exchange based on nanoparticle properties (b). Vertical lines denote ±1 SD (n = 4 for all tested samples). (*) indicates significant t-test at (P < 0.05). Bracket with a (*) represents significant one-way ANOVA at (P < 0.05).

Since protein adsorption has an important effect on cellular responses to implants, we sought to examine the protein affinity of various nanoparticles following incubation in serum containing culture media for periods of 30 minutes and 2 hours (Figure 1). Surprisingly all nanoparticles deplete similar serum protein quantities from culture media after 30 minutes (Figure 1a). However, there is a nanoparticle dependent degree of irreversible protein adsorption with additional incubation. The metal oxides and MWCNT 20-30 nm have a significant increase in the amount of protein detected in the culture media shown as a significant decrease in protein depletion. Change in protein depletion levels between 30 minutes and 2 hours time points were compared between nanoparticle types (Figure 1b). Assay results reveal that nanoparticles have a differential capacity to adsorb and to desorb proteins with additional incubation at 37°C.

### Nanoparticle uptake in different cells

**Figure 2:**
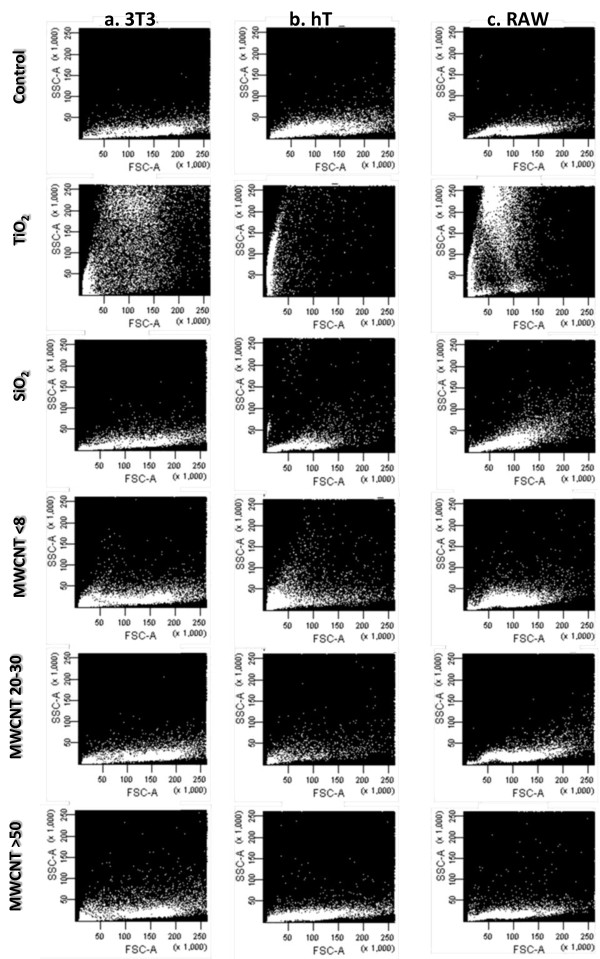
**Analysis of nanomaterial uptake**. Nanomaterial uptake by (a) 3T3, (b) hT, and (c) RAW cells. Unexposed cells were scanned to establish normal cell population. Cells exposed to nanoparticles for 3 hrs were detached from well plates and analyzed for forward scatter vs. side scatter to determine nanoparticle uptake. In the case of nanomaterial exposed cells, nanomaterial uptake/surface adsorption is reflected by increases in the side scatter of cell populations.

Since the biomaterials appear to behave differently in regard to protein adsorption and material-protein interactions have been directly linked with cellular responses [56], we compared nanoparticle uptake or attachment to cellular membranes in model cell lines using flow cytometry (Figure 2). Figures 2a, b, and 2c show the influence of nanomaterial composition and size on their uptake or their interaction with 3T3 fibroblasts, hT bronchial epithelial cells, and RAW macrophages, respectively. In all three cell types, control cells show a size distribution population with minimal side scatter, which can be related to particles in the cell or on the outside or due to changes in the organelles in the cell. Interestingly, following 3 hours exposure, we see that nanomaterials had a variable effect on the side scatter of the cells, with all three cell types exhibiting substantial uptake or interaction with TiO_2 _as evident by a large increase in side scatter. TiO_2 _exposure also produced a pronounced left shift in the cell population indicating a decrease in the cell size, due to cellular responses following nanoparticle uptake. hT bronchiolar epithelial cells exhibited the largest left shift (nanoparticle uptake-associate cell size reduction) of TiO_2 _accompanied by an increase in side scatter. On the other hand, SiO_2_, MWCNT <8 nm, MWCNT 20-30 nm and MWCNT >50 nm triggered very little to no increase in side scatter compared to controls. Control studies have shown that there was no apparent "spill-over" for all test materials in the flow cytometry analyses.

### Nanomaterial composition, concentration and size affect cell viability

**Figure 3:**
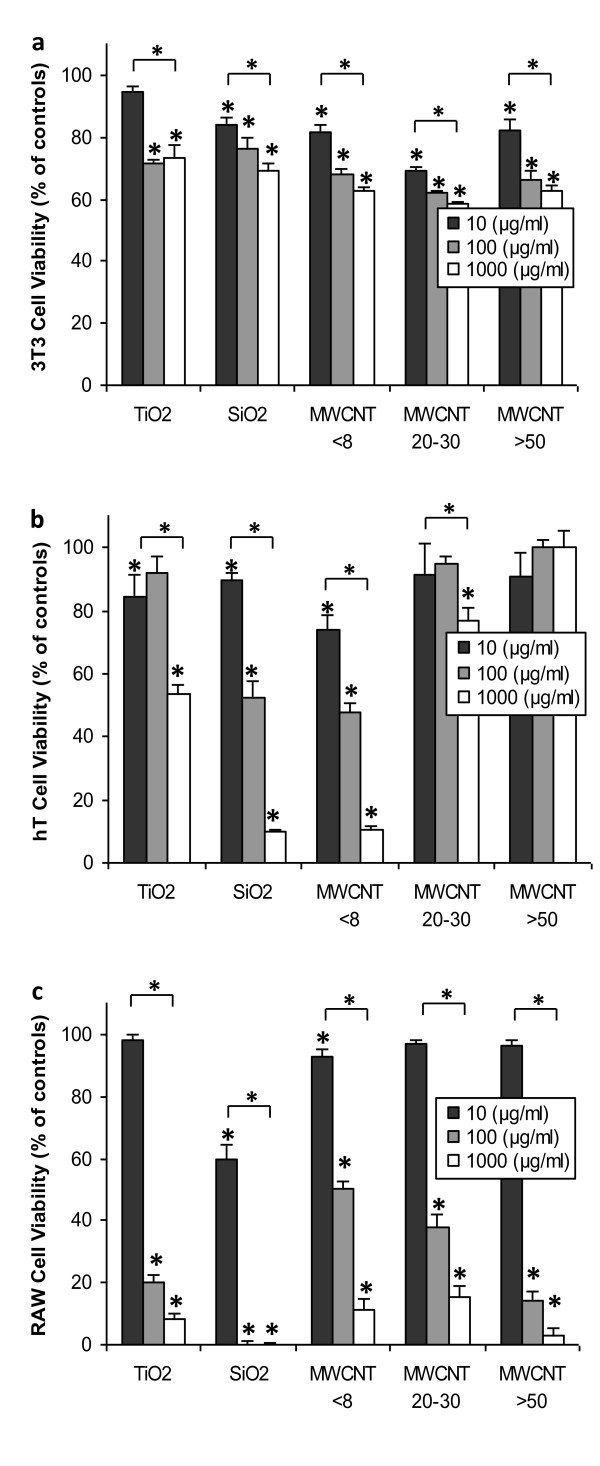
**Nanomaterial composition, concentration, and size effects on 3T3, hT and RAW cells**. The effect of nanomaterial concentration and composition on survival rates of (a) 3T3 fibroblasts, (b) hT, and (c) RAW cells at 24 hours. Cells were respectively treated with 10, 100, 1000 μg/ml of TiO2, SiO2, MWCNT <8 nm, MWCNT 20-30 nm, and MWCNT >50 nm for 24 hours. Viability was measured using the MTS assay and normalized to untreated cells. Vertical lines denote ±1 SD (n = 4 for all tested samples). Significant ANOVA test among different cell types is represented by bracket with a (*) over each data set. (*) above nanomaterial columns indicates statistically significant difference compared to untreated controls (P < 0.05).

We studied the effect of nanomaterial chemical, physical properties and concentrations on cell viability. As expected, we find that the cell toxicity is cell type, material composition and concentration dependent. Specifically, RAW macrophages are very susceptible to nanomaterial toxicity while 3T3 fibroblasts are more resistance to nanomaterial toxicity. The viability of 3T3 cells slightly decreased as the concentration of nanomaterials was increased (Figure 3a). In addition, varying the diameters of MWCNTs had minimal effect on 3T3 toxicity (Figure 3a). In hT bronchiolar epithelial cells, TiO_2 _was toxic only at a concentration of 1000 μg/ml whereas SiO_2 _and MWCNT < 8 nm were cytotoxic to these cells at both 100 and 1000 μg/ml (Figure 3b). In hT cells we found that MWCNT <8 nm were substantially more toxic than similar materials with bigger diameters (MWCNT 20-30 nm and >50 nm) (Figure 3b). In RAW macrophages, SiO_2 _exerted ~40% cell toxicity at low concentration (10 μg/ml), while other materials (TiO_2_, MWCNT <8 nm, 20-30 nm and >50 nm) showed minimal toxicity (Figure 3C). At 100 and 1000 μg/ml, all test materials were toxic to RAW macrophages (Figure 3C). Among the five nanomaterials, SiO_2 _exhibited the highest cytotoxicity to RAW macrophages. We also noticed that MWCNT >50 nm are more toxic to RAW macrophages than small diameters of materials (MWCNT <8 and 20-30 nm) (Figure 3c).

### Effect of material composition on the kinetics of cell viability

**Figure 4:**
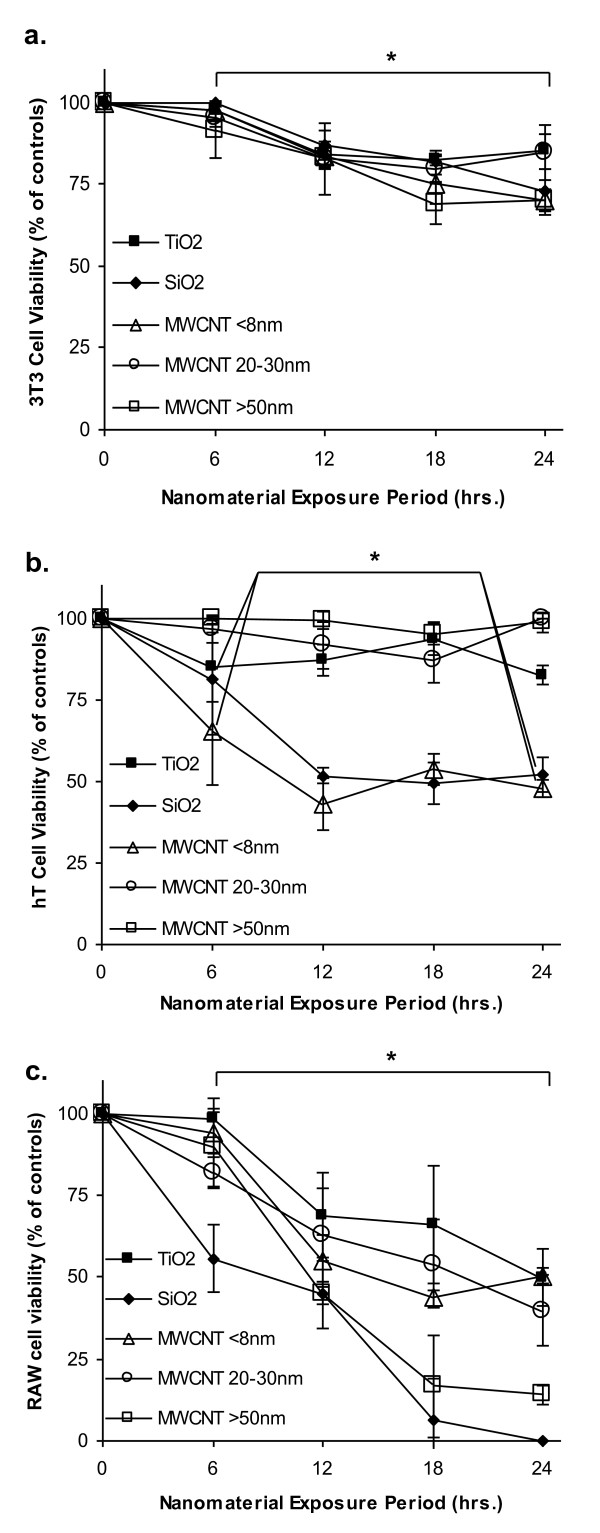
**Kinetics of cell dependent nanomaterial cytotoxicity**. Time dependent toxicity profiles for each nanomaterial group. Nanomaterials cause time-dependent drops in cell viability. Nanomaterials at 100 μg/ml were exposed to (a) 3T3, (b) hT, and (c) RAW cells for 6, 12, 18, and 24 hours. Viability was measured using MTS assay and normalized to untreated cells. Bracket with a (*) represents significant one-way ANOVA at (P < 0.05).

Although material composition affects cell viability, it is not clear whether different materials affect the kinetics of cell death. To find the answer, cell toxicity was monitored every 6 hours for 24 hours using MTS assay at a concentration of 100 μg/ml of each nanomaterial. In agreement with previous observations [27-29], we find that nanomaterials prompt the least toxicity on 3T3 fibroblast and most toxicity on RAW macrophages (Figure 4). In 3T3 fibroblasts, nanomaterials-induced cytotoxicity could be seen only after at least 6 hours of nanomaterial exposure to these cells. Furthermore, the difference in cell toxicity is minimal among test nanomaterials (Figure 4a). TiO_2_, MWCNT 20-30 nm and >50 nm nanomaterials have minimal toxicity to hT bronchiolar epithelial cells (Figure 4b). In hT cells, TiO_2 _could affect cell viability as early as 6 hours after incubation while MWCNT <8 nm showed significant cytotoxicity at 12 hour time point (Figure 4b). With RAW macrophages, SiO_2 _or MWCNT 20-30 nm associated cell death can be seen as early as 6 hours of nanomaterial exposure of these cells (Figure 4c). However, the death of RAW macrophages incubated with TiO_2_, MWCNT <8 nm or >50 nm become prominent at a later time (12 hours) (Figure 4c). These results show that both cell types and material properties affect the kinetic of cell viability.

### Nanomaterial-mediated cell toxicity - Reactive oxygen species and lysosomal membrane destabilization in nanoparticle exposed cells

**Figure 5:**
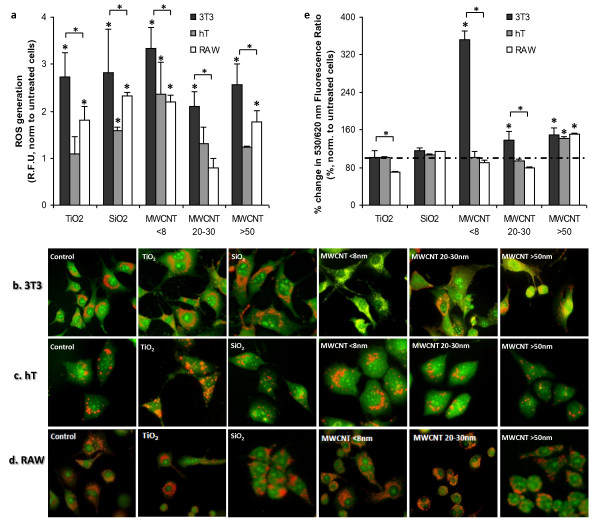
**Nanomaterial dependent ROS generation and lysosomal membrane destabilization (LMD) in 3T3, hT, and RAW cells**. (a) ROS generation was quantified using 2',7'-dichlorodihydrofluorescein diacetate (H_2_DCFDA) for untreated cells and compared to cells exposed with each test nanomaterial for 2 hours at 100 μg/ml. LMD was visualized via acridine orange staining in (b) 3T3, (c) hT, and (d) RAW cells exposed to nanomaterials at 100 μg/ml for 4 hrs. In control (healthy) cells, lysosomes can be seen as red-orange granules and cytoplasm has a diffuse green fluorescence. In cells with lysosomal membrane damage, lysosomes exhibit a shift from red-orange to a green color and overall intensity of green fluorescence is increased in these cells. (e) Quantification of LMD after 4 hours of exposure to nanomaterials at 100 μg/ml was done via acridine orange relocation technique. For each nanomaterial, bracket with a (*) indicates significant difference between different cell types by ANOVA. (*) above nanomaterial columns indicates significant difference from untreated cells by Bonferroni test.

Since material properties affect the kinetics of cell death, it is possible that mechanisms of nanomaterial-mediated cell toxicity vary depending on the composition of material each cell type interacts with. ROS generation has been suggested to be an initial cellular response to nanomaterial internalization and later cell death [13]. Since apparent nanomaterial-associated cell death can be seen as early as 6 hours following exposure, we measured the nanomaterial-mediated cell responses prior to cell death at an earlier time point - 2 hours. Specifically, we measured the production of intracellular ROS via H_2_DCFDA oxidation assay. We find significant upregulation in ROS in 3T3 cells with all nanomaterials (Figure 5a). In hT cells, we observe significant ROS generation only after exposure to SiO_2 _and MWCNT <8 nm. In RAW cells significant ROS generation was found in cells incubated with SiO_2_, TiO_2_, MWCNT <8 nm and MWCNT >50 nm. Surprisingly, our results show that, after exposed to nanomaterials, 3T3 fibroblasts produce more ROS than hT cells and RAW cells. hT cells prompted the least ROS production after incubation with TiO_2_, SiO_2 _and MWCNT >50 nm while RAW cells produced the least ROS after exposed to MWCNT 20-30 nm. We also noticed that MWCNT diameter affected the degree of ROS production in both hT cells and RAW cells (Figure 5a).

Increased ROS production has been associated with lysosomal membrane destabilization (LMD) [48]. To test this, the ability of nanoparticles and nanotubes (all at 100 μg/ml) to damage lysosomal membranes was assessed using the acridine orange (AO) relocation assay. The extent of LMD in variously treated cells can be visualized under microscopy (Figure 5b, c, d) which was then quantitatively analyzed using a fluorescence plate reader (Figure 5e). In control 3T3 cells, the lysosomes (red-orange granules) can be clearly seen (Figure 5b). 3T3 cells treated with TiO_2 _showed little or no LMD by microscopic observation. However, MWCNT <8 nm caused pronounced LMD as evidenced by the release of lysosomal contents into the cytoplasm (reduction in red and enhanced green fluorescence). Minor lysosomal damage can also be seen in some 3T3 cells treated with MWCNT 20-30 nm and >50 nm. Given the much lower ROS production in hT and RAW cells, we assumed that LMD might not be prominent in these cells. As expected, there was no detectable destabilization of lysosomal membranes observed in hT and RAW cells when exposed to TiO_2_, SiO_2_, MWCNT <8 nm and 20-30 nm by visual inspection (Figure 5c, d). Minor lysosomal damage was seen in hT and RAW cells exposed to MWCNT >50 nm (Figure 5c, d).

The extent of LMD in variously treated cells was quantified through measurement of the ratio of 530 nm and 620 nm fluorescence intensity. Control ratio was set to 100% and nanomaterial values were compared to the normalized untreated cells (Figure 5e). As observed microscopically, only MWCNT <8 nm exposed to 3T3 cells had a substantial increase in LMD, while MWCNT 20-30 nm caused only a slight increase in LMD. In agreement with visual observation, quantification of LMD in nanomaterial exposed hT cells and RAW cells affirmed no significant LMD for TiO_2_, SiO_2_, MWCNT <8 nm and 20-30 nm nanomaterials. Small degree of LMD was found in hT cells and RAW cells after incubation with MWCNT >50 nm (Figure 5e). Our results suggest that nanomaterial-associated LMD is responsible for the nanomaterial-induced toxicity in 3T3 fibroblasts, but not in hT bronchiolar epithelial cells and RAW macrophages.

### Nanomaterial-mediated cell toxicity - Mitochondrial membrane potential and caspase-3/7 activation in nanoparticle exposed cells

**Figure 6:**
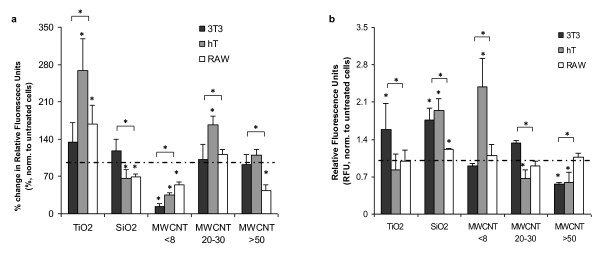
**Nanomaterial dependent Mitochondrial membrane potential (MMP) and Caspase-3/7 activation in 3T3, hT, and RAW cells**. (a) MMP was monitored following 6 hrs of nanomaterial exposure at 100 μg/ml using MitoProbe DilC_1_(5) Assay Kit. (b) Caspase activity in all three cell types was assessed using Sensolyte Homogeneous AMC Caspase-3/7 Assay Kit following 10 hours of exposure to all materials at 100 μg/ml. For each nanomaterial, bracket with a (*) indicates significant difference between different cell types by ANOVA. (*) above nanomaterial columns indicates significant difference from untreated/control cells by Bonferroni test.

To search for alternative mechanisms of nanomaterial-mediated cytotoxicity in hT bronchiolar epithelial cells and RAW macrophages, we examined the changes of mitochondrial membrane potential (MMP) in all cell lines after particle exposure. This hypothesis is supported by many recent observations that the decrements in mitochondrial membrane potential (MMP) may be responsible for apoptosis [26,48]. We find that MWCNT <8 nm caused profound loss of MMP in all cells. On the other hand, MMP reduction is found in hT cell exposed to SiO_2 _and in RAW cells incubated with either SiO_2 _or MWCNT >50 nm (Figure 6a). These results suggest that cytotoxicity of SiO_2_, MWCNT <8 nm and >50 nm in hT cells and RAW macrophages may be associated with MMP reduction.

The observed differences in ROS generation, LMD, and MMP, along with cell viability at 24 hours, led us to speculate as to whether activation of caspase 3/7 is also involved. For that, we measured the activation of caspase 3/7 in cells treated with 100 μg/ml of nanoparticles to assess activation of apoptosis. In 3T3 fibroblasts, we observe caspase 3/7 activation in cells exposed to TiO_2 _and MWCNT 20-30 nm indicated by the slight increase in detection levels (Figure 6b). In comparison to 3T3 fibroblast responses, we find that only MWCNT < 8 nm-incubated hT and MWCNT >50 nm-incubated RAW cells were able to induce significant caspase activation while no significant caspase 3/7 activation was seen in other treated hT and RAW cells (Figure 6b).

### Nanomaterial-mediated cell toxicity - Mode of nanoparticle induced cell death

**Figure 7:**
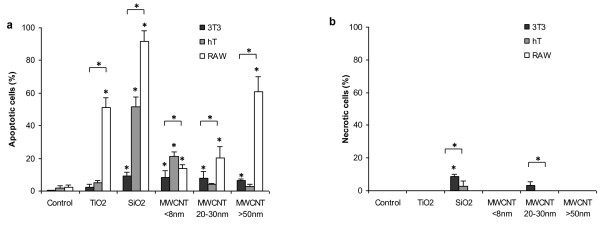
**Nanomaterial induced apoptosis and necrosis in 3T3, hT and RAW cells**. Annexin V-FITC/PI double staining was performed to distinguish 3T3, hT and RAW cells undergoing (a) Apoptosis and (b) necrosis following 20 hours of exposure to all nanomaterials at 100 μg/ml. For each nanomaterial, bracket with a (*) indicates significant difference between different cell types by ANOVA. (*) above nanomaterial columns indicates significant difference from untreated/control cells by Bonferroni test.

Since caspase-3/7 activation was found in some nanoparticle-exposed cells and caspase activation may lead to apoptosis [13], we carried out studies to study the effect of nanomaterial treatments on mode of cell death - apoptosis vs. necrosis - using annexin V/propidium iodide assay. After incubation for 20 hours, which was sufficient time to induce cell death, we found that different nanomaterials triggered different extent of cell apoptosis (Figure 7a). As anticipated, small percentages ( < 10%) of 3T3 cells incubated with SiO_2_, MWCNT <8 nm, 20-30 nm and >50 nm had undergone apoptosis. Other types of nanomaterials did not trigger apoptosis in 3T3 fibroblasts. In hT cells, we saw ~50% and ~25% apoptotic cells when exposed to SiO_2 _and MWCNT <8 nm, respectively. Interestingly, TiO_2_, MWCNT 20-30 nm and >50 nm did not cause apoptosis in hT cells (Figure 7a). In agreement with previous observations, we observed large percentages (~50, 90, and 60%) of apoptotic cells when RAW cells were incubated with TiO_2_, SiO_2 _and MWCNT >50 nm, respectively (Figure 7a). It should be noted that fewer but significant numbers, of apoptotic cells were also found in RAW cells exposed to MWCNT <8 nm and 20-30 nm. A parallel study was also carried out to determine whether necrosis plays a role in nanomaterial cytotoxicity. We find only a few nanomaterials may trigger necrotic reactions causing very small percentages of cell death (Figure 7b).

## Discussion

The purpose of this study was to examine cell dependent responses to nanomaterials of variable compositions and sizes through assessing their characteristics in aqueous solution and their ability to adsorb serum proteins from culture media, through monitoring their cellular uptake, cell viability, intracellular responses and potential mechanisms of toxicity. To distinguish these effects, we selected a model cell panel which included cells with distinctly different physiological roles, yet similar susceptibility to nanomaterial exposure whether through environmental or biomedical routes. The cell culture model was selected based on physiological relevance and nanoparticles were evaluated over a range of concentrations to additionally investigate concentration dependent effects.

Foreign particles, especially nanoparticles which have large surface area have strong tendency to agglomerate in the liquid [57] and interact with biomolecules such as proteins and DNA in biological environment [58-60]. Since these characteristics contribute to particle uptake and variable intracellular responses [59], we included these occasionally overlooked factors into our design, in accordance with recent trends in nanotoxicology publications [41,42,61]. In PBS and culture media at low concentration (10 μg/ml), among the five nanomaterials, the metal oxides displayed the highest tendency to form aggregates while MWCNT >50 nm were least inclined to form aggregates. Although the metal oxides differed in particle size, their average size in PBS was identical. Therefore it seems that a particle's surface chemistry and/or the environment, rather than size, might be the determining factor in its propensity to form aggregates in liquid as previously reported [62,63].

The size of the aggregates seemed to be concentration dependent and extent of nanomaterial aggregation was identical in both PBS and cell culture media, with aggregate size slightly larger in cell culture media. Since the presence of monovalent and divalent cations has been shown to cause nanomaterials to aggregate, the observed similarity could be because of similarity in ionic strength between these solutions [63]. The presence of serum proteins did not significantly affect the size of aggregates, but since nearly all foreign materials adsorb a layer of host protein [59] we examined the level of protein adsorption on these different nanomaterials. Our results are consistent with this phenomenon and are in agreement with previous reports on metal oxides and carbon nanotubes [41,43,61] which found that all nanomaterials adsorb media components such as Ca^2+ ^and serum proteins, which then affects their agglomeration and size distribution. It has been hypothesized that protein adsorption decreases with increasing size [41], a trend which we were unable to show in this study and is likely more related to the particle geometry and charge than size alone. Although all nanomaterials initially adsorbed similar amount of serum from the media, the total amounts of adsorbed protein decreased with time and the extent of protein desorption was material dependent. This might be one of the factors leading to varied intracellular responses and toxicological outputs because once proteins are adsorbed onto nanoparticle surfaces they might undergo conformational changes [58]. This could then cause exposure of new epitopes that may accidentally be recognized by signalling proteins inside cells and trigger signalling pathways [58]. Such processes can also affect a nanomaterial's association with cellular membrane and uptake in cells [42,62].

The flow cytometry uptake studies showed that the nanomaterial affected the side scatter, which can be related to nanomaterial uptake or its presence on the membrane of the cell or due to changes in the organelles within the cell. This is based on not only their size and geometry, but also their protein adsorption properties. TiO_2 _nanoparticles caused the greatest increase in side scatter in all cells followed by MWCNT <8 nm and MWCNT >50 nm while MWCNT 20-30 and SiO_2 _caused the least shift in side scatter by all cells. Although the cause for high cellular uptake/interaction of TiO_2 _nanoparticles by cells has yet to be determined, it is possible that agglomerated TiO_2 _nanoparticles form particle clusters and thus accumulated higher amount of adsorbed protein compared with larger size particles, such as SiO_2 _nanoparticles, as indicated in a recent report [57]. Additionally, we observed that the geometry of nanomaterials also appeared to affect their uptake/interaction in all three cells, especially with the ultra-small TiO_2 _nanoparticles and MWCNT <8 nm. This is in agreement with recent report which showed that rod shaped nanoparticles had lower uptake compared to spherical shaped nanoparticles [64]. We have also noticed that different cells exert varying extent of particle uptake/interaction. Amongst all the nanomaterials investigated, the lowest uptake/interaction of SiO_2 _and MWCNT 20-30 nm was observed in 3T3 fibroblasts with a similar degree of side scatter as well. However, in hT bronchiolar epithelial cells, MWCNT >50 uptake/interaction was the least while SiO2 and MWCNT 20-30 nm exhibited slightly higher, yet still similar, amount of uptake/interaction. In RAW cells, however, MWCNT 20-30 nm appeared to interact or was taken up the least by these cells. We validated these responses prior to any particle-induced toxicity studies as many studies investigate nanoparticle exposure in the absence of serum, a distinctly non-physiological situation, which may affect nanoparticle uptake in cells and hence toxicity. Furthermore, flow cytometric analyses on concentration dependent change in side scatter were carried out at the other two concentrations, 10 μg/ml and 100 μg/ml. We found that increased nanomaterial exposure resulted in proportional increases in particle uptake in agreement with other studies on metal oxides, ceria and polymer nanoparticles [45,57,65]. As a result, this may lead to contradictory cellular responses and dramatically alter the mechanism of uptake and toxicity [66]. Nanoparticle-induced cell toxicity also varied based on particle composition, size and the cell type it was exposed to; however, the particle uptake did not correlate with its cytotoxicity.

Consistent with many previous observations [49], we observe that toxicity increases dependent on concentration. RAW macrophages appear particularly susceptible to higher concentrations, more so than 3T3 or hT cells regardless of particle composition or size. This is likely related to the physiological function of macrophages [67]. It is well established that macrophages readily phagocytose non-targeted nanoparticles at a very high rate [65,68], and are more capable of uptake than other stromal cell types. The avid uptake of nanoparticles by these cells makes them more susceptible to particle overload and cell death, especially in the case of nanoparticles with non-spherical geometries - such as MWCNT- which take longer to expel than spherical nanoparticles [69,70]. In addition, macrophage interactions with nanoparticles may lead to cytolysis, resulting in the release of inflammatory mediators which increase systemic responses to the foreign material [65,70]. As such, these cell dependent processes may explain higher toxicity levels seen in RAW macrophages at higher concentrations investigated. In addition, possibly due the physiological functions of fibroblasts and epithelial cells, these cells appear to be less sensitive to increases in concentration in comparison to RAW macrophages. This may be due to intracellular mechanisms which restrict continuous nanoparticle uptake due to saturation [71-73].

In addition to nanomaterial composition, size and concentration, the influence of cell type is of paramount importance in nanomaterial toxicity as highlighted in other recent investigations in cell vs. cell comparisons [49]. Both 3T3 fibroblasts and hT epithelial cells only showed susceptibility to TiO_2 _at elevated concentrations where viability fell to near 50%. Though responses were similar, TiO_2 _was able to induce substantially higher levels of ROS in 3T3 than in hT cells. ROS generation in 3T3 cells exposed to nanoparticles has also been reported by many other groups [14,74]. Reduced ROS in hT bronchial epithelial cells might be associated with the presence of metallothionein (MT), a cysteine-rich metal-binding and detoxification protein which protects against oxidative damage and is upregulated in lung cells under oxidative stress [75-82]. However, TiO_2 _had a much more prominent cytotoxic effect on RAW macrophages, potentially related to cell properties as previously mentioned as well as to the tendency of TiO_2 _to promote ROS generation after uptake in RAW macrophages [83]. However, MWCNT < 8 nm were generally more toxic to all cells (similar diameter, cylindrical vs. spherical geometry) than TiO_2_. Several investigations have reported increased toxicity of MWCNT compared to other nanoparticle types [84,85], and may related to geometry and agglomeration [86] and subsequent mechanism of uptake followed by downstream cellular responses.

To what degree does size dictate the cellular response? It has been suggested that the smaller a nanomaterial's dimensions the greater its toxicity [87]. In addition, geometrical configurations have been shown to affect the response [88]. However, our results demonstrate the importance of both cell type and composition regarding size considerations. For example, with RAW macrophages at 100 μg/ml TiO_2 _nanoparticle toxicity is less than MWCNT <8 nm which are of similar size. To our surprise, however, the cylindrical shaped but larger diameter MWCNT 20-30 nm had a cell specific response, with low toxicity to hT cells, moderate for 3T3 cells, and high toxicity to RAW macrophages. Interestingly, the toxicity of 3T3 and hT to MWCNT 20-30 nm only slightly changes with increasing concentration, while RAW macrophages have a drastic reduction in viability as concentration increases. This may be due to reduced fibroblast and epithelial cell interaction with the certain nanoparticle formulations, where the macrophages may have an enhanced ability to interact with the same nanomaterials based on innate physiological functions [49]. This hypothesis is supported by our flow cytometry data, which suggest that TiO_2 _and MWCNT < 8 nm are able to more quickly and efficiently be taken up into cells than the larger MWCNT 20-30 nm. Based on these results, it appears that material properties and geometry of nanomaterials may have a more prominent effect on cellular responses than size alone. However, larger discrepancies in size (micro vs. nano scale) and shape (spherical vs. rod) between materials of the same composition will likely yield size/shape dependent in vitro and in vivo cellular responses as shown in previous studies [88-93].

Finally we assessed the potential mechanisms of toxicity by investigating the ability of nanoparticles of different size and composition to cause lysosomal membrane destabilization, to reduce mitochondrial membrane potential and to activate caspase 3/7. Various toxicity studies have shown that nanomaterial-induced ROS generation causes lysosomal membrane destabilization, DNA damage and mitochondrial membrane potential; leading to increases in the activation of p53, caspase-3 and caspase-7, and eventual cell death via apoptosis [13,15,17,22,94,95]. Our results suggest that nanomaterials may cause cell death via different cytotoxicity pathways as shown in Table 3. Specifically, 3T3 fibroblasts are most resistant to nanomaterial toxicity among the three cells that were tested. The cytotoxicity of TiO_2 _and SiO_2 _nanomaterials to 3T3 cells is likely mediated by ROS generation followed by caspase 3/7 activation. It should be noted that both TiO_2 _and SiO_2 _nanomaterials increase ROS production without triggering lysosomal membrane destabilization, although increased ROS have been shown to cause lysosomal destabilization and rupture leading to apoptosis [48,96]. Furthermore, regardless of MWCNT diameter, MWCNT-mediated fibroblast toxicity is associated with ROS production, LMD, and eventual cell death via apoptosis (Table 3). This sequence of events is supported by recent observations that a high degree of lysosomal membrane destabilization early after nanoparticle exposure may lead to excessive leakage of lysosomal contents into the cytoplasm causing cell death [97-99]. Interestingly, MWCNT < 8 nm-mediated reduction of mitochondrial membrane potential and MWCNT 20-30 nm-associated caspase 3/7 activation may also contribute to nanotoxicity in 3T3 fibroblasts.

**Table 3:** Summary of Cellular Responses to Nanomaterials.

Relative degree of cell responses	3T3 fibroblasts					hT bronchial epithelial cells					RAW macrophages				
	**TiO**_**2**_	**SiO**_**2**_	**MWCNT**			**TiO**_**2**_	**SiO**_**2**_	**MWCNT**			**TiO**_**2**_	**SiO**_**2**_	**MWCNT**		
												
			**< 8**	**20-30**	**> 50**			**< 8**	**20-30**	**> 50**			**< 8**	**20-30**	**> 50**
									
Cytotoxicity	+	+	+	+	+	+	++	++	+	-	+++	+++	++	++	+++
ROS	+	+	+	+	+	-	+	+	-	-	+	+	+	-	+
LMD	-	-	+++	+	+	-	-	-	-	+	-	-	-	-	+
MMP	-	-	++	-	-	-	+	++	-	-	-	+	+	-	+
Caspase 3/7	++	++	-	+	-	-	++	+++	-	-	-	-	-	-	-
Apoptosis	-	+	+	+	+	-	+++	++	-	-	+++	+++	+	+	++
Necrosis	-	+	-	-	-	-	-	-	-	-	-	-	-	-	-

The relative tendency of each nanomaterial to induce cellular responses related to cytotoxicity, ROS, LMD, MMP, and caspase activation were tabulated along with mechanistic evaluation of toxicity mechanisms through Annexin V-FITC and PI staining at 20 hours. (+) indicates a significant increase in the intracellular event while (-) indicates that the material did not induce a harmful response. Similarly a (++) twice the response compared to (+) and (+++) three times as much.

Both SiO_2 _nanomaterials and MWCNT < 8 nm exert substantial cytotoxicity to hT bronchiolar epithelial cells. Rather uniquely, despite distinct chemical and physical properties, both types of nanomaterials caused toxicity of hT cells via identical sequence of events including ROS production, mitochondrial membrane potential loss, caspase 3/7 activation, and apoptosis (Table 3). In the case of nanotoxicity to RAW macrophages, there is a good relationship between the percentage of cell death and of apoptotic cells (Table 3). Lysosomal membrane destabilization was only found in RAW cells treated with MWCNT >50 nm. Mitochondrial membrane potential loss does not correlate with cytotoxicity and can only be found in cells incubated with SiO_2_, MWCNT < 8 and > 50 nm. Differing from 3T3 cells and hT cells, caspase 3/7 activation played an insignificant role in nanomaterial-associated cytotoxicity of RAW macrophages (Table 3). RAW macrophages have previously been shown to be susceptible to caspase activation and apoptosis after polystyrene nanoparticle uptake [49]. It is possible that nanomaterial-associated caspase activation in RAW cells is unique to polystyrene materials. This provides an illustration of the fact that nanoparticles of different composition and size can generate different degrees of intracellular responses which may not directly correlate with other intracellular responses and mechanisms of cytotoxicity.

Since nanomaterial exposure can occur through a variety of routes depending on the environmental or medical nature of the exposure, several different cell types could potentially be exposed. For instance, TiO_2 _instilled into the trachea has been shown to be highly pro-inflammatory and induce fibrosis. In this model, TiO_2 _delivered at 1000 μg/ml induced only moderate toxicity to bronchiolar epithelial cells. However, lower concentrations may exert stronger cytotoxicity to pulmonary macrophages. We believe this highlights the need for studies such as this, which may provide transitions to animal models with knowledge of cell susceptibilities and potential routes of toxicity.

## Conclusions

Overall, nanomaterial size and composition plays a distinct role in the cellular response. In addition, this response is variable between cell types and is likely related to the physiological function of the cell types. We also verified that the same material can cause different intracellular responses and potential mechanism of toxicity depending on the exposed cell type. We believe these findings both highlight the importance of analyzing the effects of nanoparticles in the most relevant exposure model and support the idea that nanoparticle engineering strategies should be focused on the potential cell types which might be normally exposed to the particles. The ability to engineer nanoparticles that minimize cytotoxicity to a range of potentially exposed cells will assist future nanoparticle development and safety. Finally, we find that potential toxicity mechanisms of nanoparticle interactions with tissue and blood cells may differ drastically from external barrier cells and warrants further investigation.

## Competing interests

The authors declare that they have no competing interests.

## Authors' contributions

SKS carried out the 6, 12, 18 and 24 h MTS assays, ROS, LMP, MMP and Caspase-3/7 assays, nanomaterial uptake studies and AnnexinV/PI staining, participated in protein adsorption studies and in the design of the study, compiled and analyzed the data, performed the statistical analyses and drafted the manuscript. PTT carried out the DLS measurements, protein adsorption studies, 24 h MTS assay, ROS, LMP, MMP and Caspase-3/7 assays, performed statistical analyses and drafted the manuscript. DB cultured and maintained the colonies of the cells, participated in cytotoxicity studies and nanomaterial uptake studies. JWE participated in the design of study and manuscript drafting. LT conceived of the study, and participate in its design and coordination and participated in manuscript drafting. All authors read and approved the final manuscript.
